# Diastereo- and enantioselective preparation of cyclopropanol derivatives

**DOI:** 10.3762/bjoc.15.71

**Published:** 2019-03-21

**Authors:** Marwan Simaan, Ilan Marek

**Affiliations:** 1 The Mallat Family Laboratory of Organic Chemistry, Schulich Faculty of Chemistry, Technion-Israel Institute of Technology, Haifa, 32000, Israel

**Keywords:** carbocupration, cyclopropanol, cyclopropene, regioselectivity, stereoselectivity

## Abstract

The diastereoselective carbocupration reaction of alkoxy-functionalized cyclopropene derivatives, followed by a subsequent trapping of the resulting cyclopropylmetal species with an electrophilic source of oxygen (oxenoid) afforded various tetrasubstituted cyclopropanol derivatives in high diastereo- and enantiomeric ratios. Similarly, the enantioselective copper-catalyzed carbomagnesiation/oxidation (or amination) sequence on achiral nonfunctionalized cyclopropenes provided the desired cyclopropanol (and cyclopropylamine) derivatives in excellent diastereo- and enantiomeric excesses.

## Introduction

**Scheme 1:**
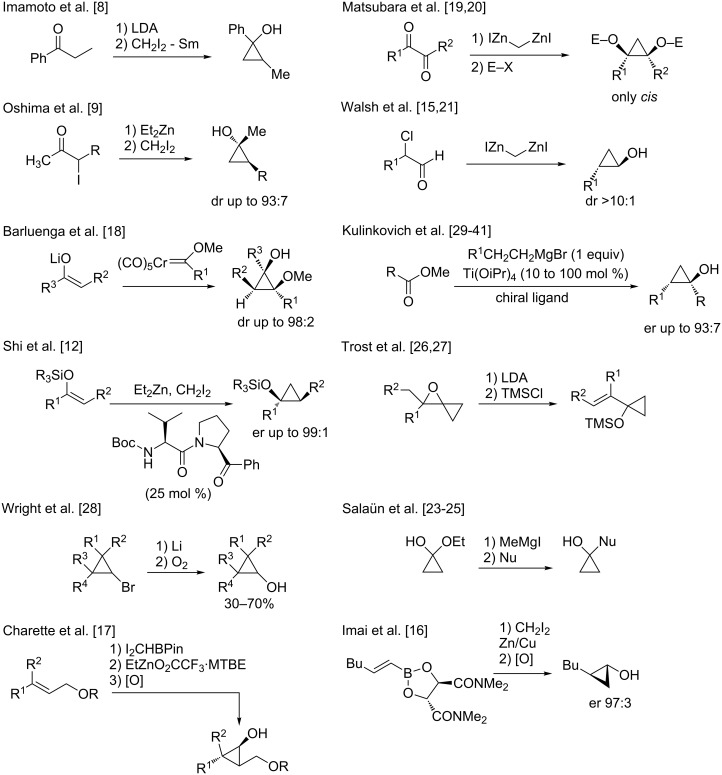
Various strategies leading to the formation of cyclopropanols.

The highly strained structure and bonding properties of cyclopropyl rings have constantly fascinated successive generations of chemists. These small carbocycles are known to have high ring strain (27.5 kcal/mol) and limited degrees of freedom, making them very attractive substrates for various chemical transformations [1]. The cyclopropane subunit is also present in many biologically important compounds such as pheromones, fatty acid metabolites, unusual amino acids and possess interesting herbicidal, insecticidal, antibiotic, antibacterial, antifungal, antiviral and antitumor activities [2]. For these reasons, cyclopropanes have been extensively studied and numerous approaches have been described for their preparation [3]. Among all possible three-membered ring subunits that have been reported, cyclopropanols have recently attracted renewed attention as they are not only contained in many natural products but they are also important precursors in the synthesis of various biologically active molecules and pharmaceuticals such as antidepressants, antiviral and antibacterial drugs [4]. Cyclopropanols and their derivatives are considered to be carbocyclic homologues of enols presenting similar chemical properties which are caused by the unsaturated character of the cyclopropyl ring. Although cyclopropanols are usually less reactive than enols and enolates, their chemical properties are somehow more diverse as they undergo useful transformations either with preservation or rupture of the cyclic structure [5]. Several reliable approaches to produce cyclopropanols have been reported in the literature (Scheme 1) [6] but a number of challenges still exist particularly for the stereoselective preparation of cyclopropanols of high structural complexity and substitution pattern. Since the first synthesis of cyclopropanol by Cottle [7], the most popular methods for the preparation of cyclopropanols rely on the transformation of enolates [8–9], silyl enol ether [10–12], vinyl borane [13–17], Fischer carbene addition [18], addition of nucleophiles to carbonyl groups [19–25], elimination [26–27], oxidation [28] or on the Kulinkovich reaction [29–41] as summarized in Scheme 1. However, and despite the increasing and justified popularity of all of these methods, the short summary described in Scheme 1 emphasizes an intrinsic problem: a different strategy is required for every cyclopropanol and cyclopropylamine that one needs to prepare, limiting the rapid structural diversification which is usually essential for biological studies. From this rapid tour d’horizon, it is clear that if one could design a stereoselective synthetic pathway to afford polysubstituted cyclopropanols (or cyclopropylamines) potentially bearing several diastereo- and enantiomerically enriched adjacent stereogenic centers, including quaternary carbon stereocenters, as single diastereo- and enantiomer from a simple precursor, it would certainly provide an additional and useful entry to the synthesis of these heterosubstituted three-membered rings.

## Results and Discussion

**Scheme 2:**
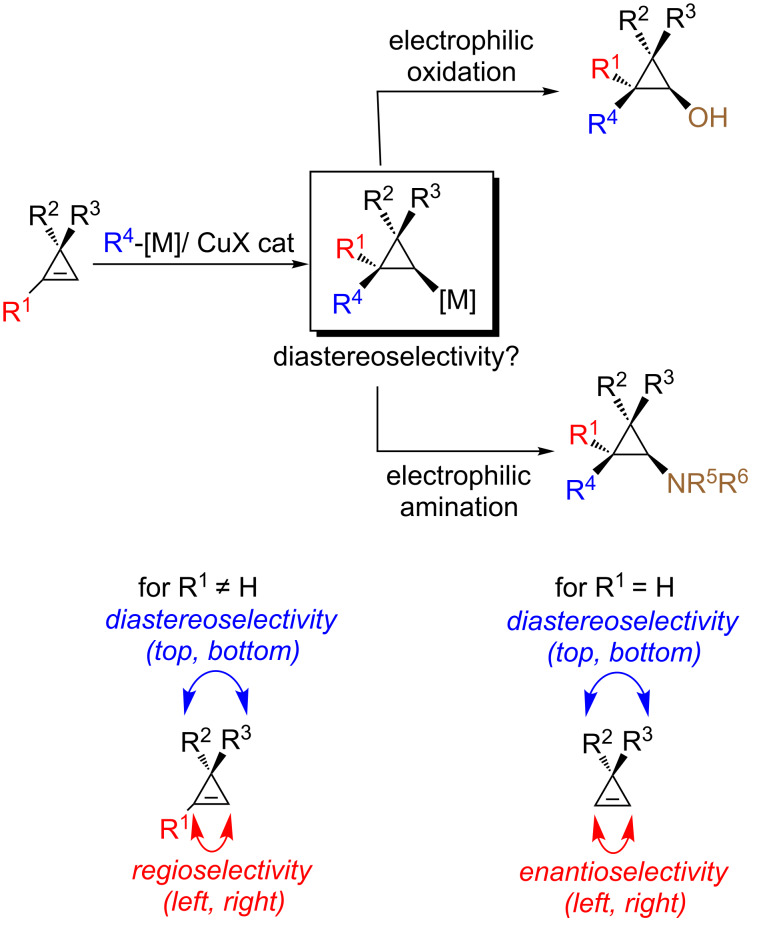
General approach to the preparation of cyclopropanol and cyclopropylamine derivatives.

To reach these goals, we are describing herein the diastereo- and enantioselective carbometalation reaction of cyclopropenes to provide cyclopropylmetal species. By subsequent stereoselective reactions of the cyclopropylmetal species with an electrophilic source of oxygen (or nitrogen), cyclopropanols (and cyclopropylamines) should be easily accessible (Scheme 2).

**Figure 1:**
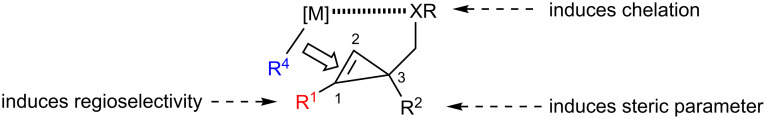
Prerequisite for a regio- and diastereoselective carbometalation.

In order to assemble both cyclopropanols and cyclopropylamines from a single and unique precursor in a single-pot reaction double facial selection by the catalyst is required: (i) regioselectivity when R^1^ is different to H and enantioselectivity when R^1^ is equal to H (left or right) and (ii) diastereotopic face selection (top or bottom) as described in Scheme 2. Since the pioneering addition of a carbon–metal bond (carbometalation) across the double bond of cyclopropenes [42], a very large number of groups have reported the addition of organometallic species demonstrating the generality of this approach for the preparation of cyclopropanes [43–63]. To achieve good diastereoselectivity during the carbometalation reaction, few conditions needed to be fulfilled in the design of the starting cyclopropenyl ring (Figure 1):

1. The presence of a coordinating group (such as an oxygen) at the C_3_ position is crucial for the selective facial addition of the incoming organometallic species.
2. The presence of a bulky substituent (alkyl or aryl) at the opposite face at the C_3_ position might equally be important as it might induce an additional steric parameter leading to a potentially more selective carbometalation reaction.
3. The substitution pattern on the double bond needs to be addressed carefully as it plays an important role in the control of the regioselectivity of the reaction pathway. An alkyl group on the C_1_ position of the cyclopropene should control the regioselectivity of the addition of the organometallic species to give the more stable secondary cyclopropylmetal species (metal at C_2_).

**Scheme 3:**
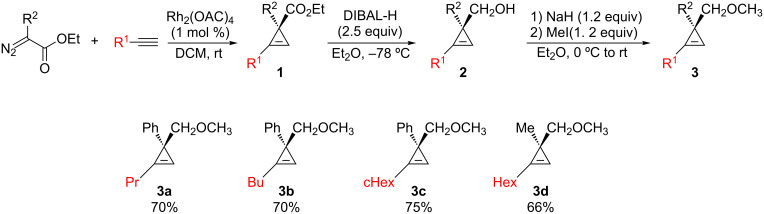
Preparation of cyclopropenyl methyl ethers **3a–d**.

We therefore decided to start our research with cyclopropanes bearing an electron-rich methoxy group on one side of the ring and a phenyl or methyl substituent on the opposite side, respectively. Following reported methods from the literature [64–67], cyclopropenes **3** were easily prepared through the well-known Rh^II^-catalyzed decomposition of diazo esters in the presence of alkynes to give cyclopropenyl esters **1**. Reduction of **1** using DIBAL-H afforded cyclopropenyl alcohols **2** and subsequent protection of the primary alcohols gave **3** in good yields (Scheme 3).

**Scheme 4:**
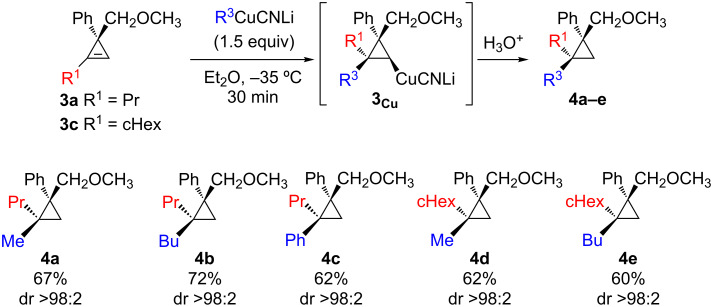
Regio- and diastereoselective carbocupration of cyclopropenyl methyl ethers **3a**,**c**.

First, we checked that the carbometalation reaction was regio- and diastereoselective by addition of lower order cyanocuprate, easily obtained from the corresponding organolithium and a stoichiometric amount of CuCN (Scheme 4) [68–70].

**Scheme 5:**
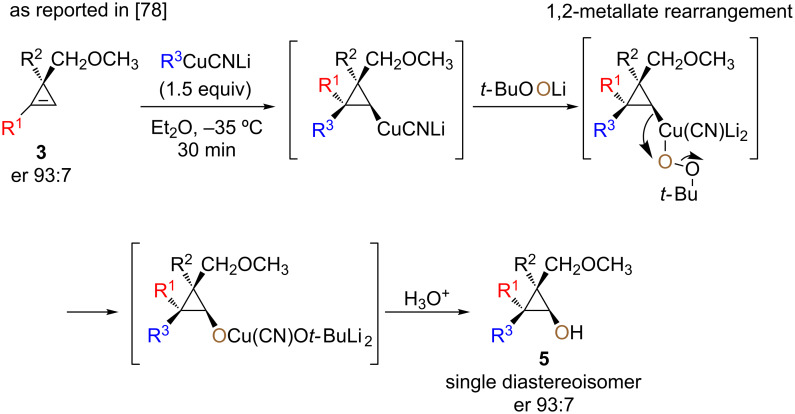
Diastereoselective formation of cyclopropanols.

The addition reaction proceeds similarly for the addition of alkyl- or arylcuprate (**4a**,**b** and **4c**) as well as for the addition on cyclopropene possessing either a primary or secondary alkyl group at the vinylic carbon center (**4a–c** and **4d**,**e**). Having in hand diastereoisomerically pure and configurationally stable cyclopropylcopper species **3_Cu_**, we next turned our attention to their stereoselective oxidation reaction (Scheme 5). Considering electrophilic oxidation processes of organometallic species, molecular oxygen seems to be the most obvious choice due to its abundancy and low cost. Nevertheless, the reaction of molecular oxygen with a organocopper species usually proceeds through single-electron transfer to dioxygen, leading to either a loss of stereoselectivity, degradation of the organocopper or to the formation of dimer as major products [71]. Therefore, it was clear that a different approach for the oxidation process was needed. Oxenoid, possessing the general structure M–O–LG, with a metal and a leaving group connected to an oxygen atom, have been shown to be an excellent electrophilic oxygen source for nucleophilic organometallic species [72]. Since the original discovery of Müller and Töpel of lithiated peroxides [73], several studies have been reported on the reactivity of oxenoids [74–77], indicating that the reaction of a nucleophile with oxenoid proceeds through an S_N_2 process [74]. Following the carbocupration of cyclopropene **3** into cyclopropylcopper species, the subsequent oxidation with the amphiphilic lithiated hydroperoxide *t-*BuOOLi (oxenoid), simply generated by deprotonation of *t-*BuOOH with *n-*BuLi, led to the copper alkoxide, as anticipated, without the formation of free radical intermediates. As already reported [78], the expected 2,2,3,3-tetrasubstituted cyclopropanols **5** were obtained as single diastereoisomers (Scheme 5).

**Scheme 6:**
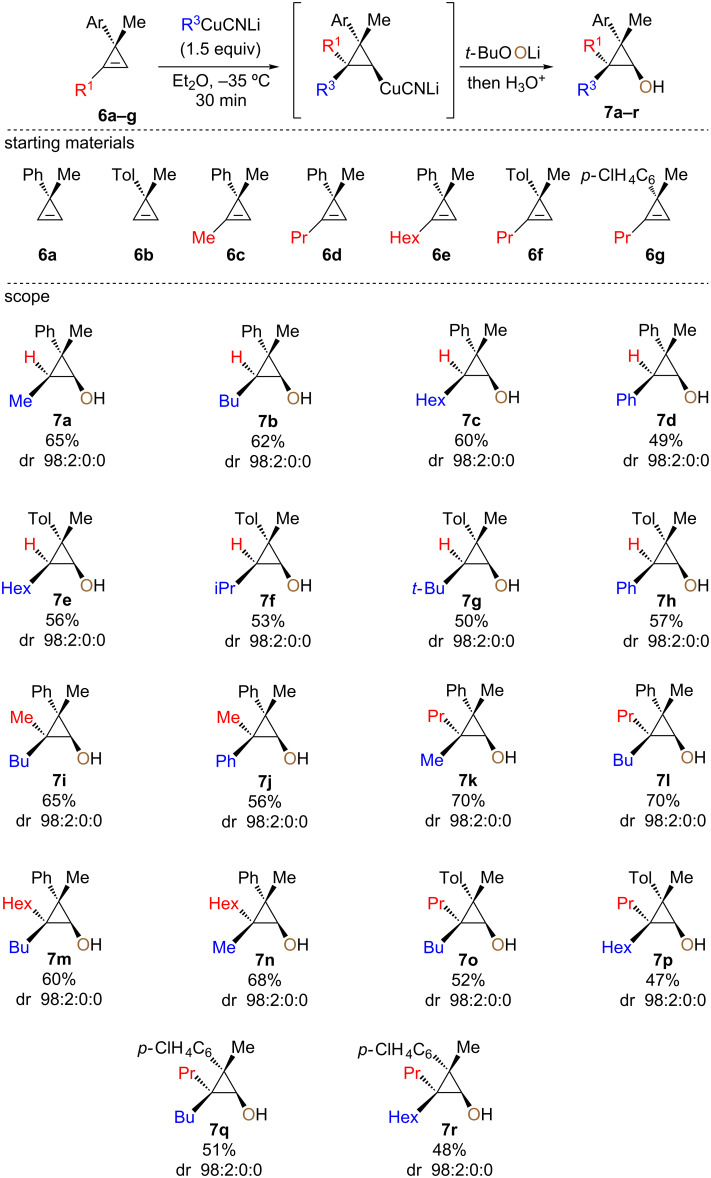
Diastereoselective carbometalation/oxidation of nonfunctionalized cyclopropenes **6**.

The reaction proceeded for all R^1^ and R^2^ groups tested and determination of the stereochemistry confirmed that the oxidation reaction proceeds with pure retention of configuration at the metalated center (intramolecular S_N_2 reaction or 1,2-metalate rearrangement) [78–80]. It should be noted that when the same sequence of diastereoselective carbometalation/oxidation was performed on cyclopropenyl ester **1**, the in situ-formed donor–acceptor cyclopropanol undergoes a selective ring-opening to provide the acyclic product possessing a quaternary carbon stereocenter [57]. As the enantioselective synthesis of the cyclopropenylmethyl ether **3** was easily achieved in high enantiomeric ratio (er 93:7, Scheme 5 [78,81–82]), the subsequent combined diastereoselective carbometalation reaction and oxidation gave the enantiomerically enriched cyclopropanols **5** as unique diastereoisomer with the same enantiomeric ratio as the starting material (Scheme 5, dr 98:2:0:0, er 93:7). Having established the optimized reaction conditions for the preparation of diastereomerically pure 2,2,3,3-tetrasubstituted cyclopropanol derivatives **5**, we were interested to expand the scope of this transformation and include different types of cyclopropene precursors. We concentrated our efforts on the reaction of diversely substituted 3-methyl-3-arylcyclopropenes **6**. In this case, as there is no coordinating functionality to dictate the facial selectivity, the control of the diastereoselectivity may be more challenging. Performing the same carbocupration/oxidation sequence on 3-methyl-3-phenylcyclopropene **6a**,**b** (R^1^ = H), we were pleased to observe that trisubstituted cyclopropanols **7a–h** were obtained in good yields with excellent diastereoselectivities (Scheme 6).

The addition of primary, secondary and tertiary alkylcuprates or arylcuprates proceeded smoothly to give the desired cyclopropanols (**7a–h**) after hydrolysis. In all cases, the easily prepared cyclopropanol derivatives were obtained with the methyl, the incoming organometallic and the alcohol in the *syn*-orientation which was determined through comparison of the hydrolyzed carbometalated products with compounds already described in the literature [83]. To further increase the structural complexity of the final cyclopropanols, we also tested the reaction on nonfunctionalized trisubstituted cyclopropenes (**6c–g**). Addition of primary alkyl- or arylcuprates followed by the oxidation of the cyclopropylcopper species proceeded equally well and gave the corresponding cyclopropanols possessing two adjacent quaternary carbon stereocenters (**7i–r**) in good yields and excellent diastereomeric ratios. Here again, the methyl, the alkyl group from the organometallic and the alcohol in the resulting cyclopropanols are *syn*-oriented as previously observed. As reported in a different context, the nature of the two substituents on the cyclopropene rings could be changed without drastically altering the selectivity of the reaction [83,91].

**Scheme 7:**
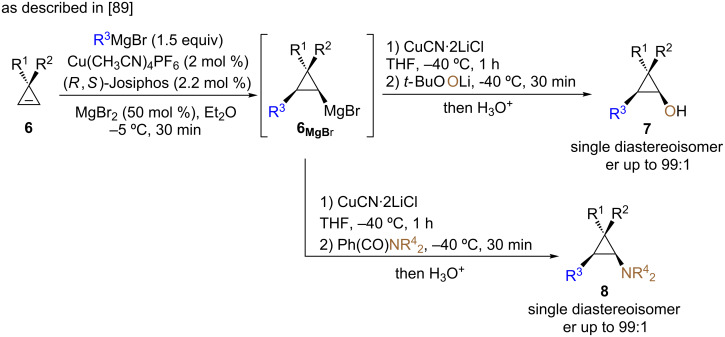
Preparation of diastereoisomerically pure and enantioenriched cyclopropanols and cyclopropylamines.

We were also interested to develop the access to non-racemic unfunctionalized cyclopropanols. Based on the pioneering work of Lautens [62], Nakamura [60], Fox [61], Gevorgyan [51] and Tortosa [84], we anticipated that an enantioselective copper-catalyzed carbometalation reaction [83,85] would be an ideal solution to access the desired polysubstituted enantioenriched cyclopropanols. As reported, the copper-catalyzed diastereo- and enantioselective carbomagnesiation reaction of cyclopropenes **6** was easily achieved in the presence of (*R*,*S*)-Josiphos (2.2 mol %, Scheme 7). Having in hand, diastereoisomerically pure and enantiomerically enriched cyclopropylmagnesium species **6_MgBr_**, the selective oxidation reaction of the copper species, resulting from a transmetalation reaction, was similarly achieved by reaction with oxenoid [71,79–80,86–89]. In all cases, cyclopropanols **7** were obtained as single diastereoisomer (dr 98:2:0:0) with excellent enantiomeric ratios (er up to 99:1, Scheme 7). Following the same concept of copper-catalyzed diastereo- and enantioselective carbomagnesiation reaction of cyclopropenes **6** followed now by a selective electrophilic amination reaction, a powerful entry to cyclopropylamines as single diastereoisomer and in excellent enantiomeric ratios could also be achieved (Scheme 7) [89]. However, the enantioselective and catalytic copper-catalyzed carbomagnesiation reaction gave poor enantiomeric ratios for the addition of vinyl, aryl and allyl groups and alternative strategies have been recently developed in our research group [90–92].

## Conclusion

In conclusion, we have successfully merged the regio- and diastereoselective carbocupration reaction of alkoxy-functionalized cyclopropenes with electrophilic oxidation of the resulting cyclopropylcopper species to afford 2,2,3,3-polysubstituted cyclopropanol derivatives bearing two adjacent quaternary stereogenic centers in a single pot operation. The simple preparation of enantiomerically enriched cyclopropene afforded the corresponding cyclopropanols in high enantiomeric excess. This transformation was then applied to unfunctionalized diversely substituted cyclopropenes. Using the catalytic and enantioselective carbometalation reaction of unfunctionalized cyclopropenes followed by an electrophilic oxidation reaction, polysubstituted cyclopropanols were obtained as single diastereoisomer with high enantiomeric ratios. In all cases, the configurationally stable cyclopropylmetal species reacted with retention of configuration with those electrophiles opening a new approach to *O*-heterosubstituted cyclopropyl rings.

## Experimental

### General procedure for the carbocupration reaction of **3a**,**c** with RCuCNLi

To a suspension of CuCN (1.5 equiv) in 8 mL of Et_2_O was added alkyllithium dropwise at −35 °C (2 equiv). The resulting mixture (pale yellow in case of MeLi and PhLi and dark brown in case of *n-*BuLi and *n-*HexLi) was allowed to stir for 30 min. Cyclopropene **3a**,**c** (1 equiv in 2 mL/mmol of Et_2_O) was added at that temperature and the reaction mixture was stirred until TLC shows complete consumption of the starting material (eluent hexane/EtOAc 9:1, ca. 30 min). The reaction was then quenched with an aqueous solution of NH_4_Cl/NH_4_OH (2:1). The aqueous layer was extracted twice with EtOAc and the combined organic phases were washed with brine, dried over MgSO_4_, filtered, and concentrated under reduced pressure. The crude mixtures were then purified by flash chromatography using pentane/diethyl ether as eluent.

### General procedure for the combined carbocupration/oxidation sequence

The reaction was performed on a 1 mmol scale. To a suspension of CuCN (2 equiv) in 8 mL of Et_2_O was added alkyllithium dropwise at −35 °C (2 equiv/2 mmol). The resulting mixture (pale yellow in case of MeLi and PhLi and dark brown in case of *n-*BuLi and *n-*HexLi) was allowed to stir for 30 min. Cyclopropene **6a–g** (1 equiv/1 mmol in 2 mL of Et_2_O) was added at that temperature and the reaction mixture was stirred until TLC shows complete consumption of the starting material (eluent hexane/EtOAc 9:1, ca. 30 min). The oxenoid was prepared in a different flask by slowly adding *n-*BuLi (1.2 equiv) to a solution of *tert-*butyl hydroperoxide (2 equiv) in THF (5 mL/2 mmol) at −80 °C. After 30 min at −80 °C, the resulting *t-*BuOOLi was transferred to the organocopper dropwise at −78 °C via a cannula. The mixture (orange to brown) was stirred at this temperature until disappearance of the cyclopropylcopper species (followed by TLC, eluent hexane/EtOAc 9:1, ca. 30 min). The reaction was then quenched with an aqueous solution of NH_4_Cl/NH_4_OH (2:1). The aqueous layer was extracted twice with Et_2_O and the combined organic phases were washed with brine, dried over MgSO_4_, filtered, and concentrated under reduced pressure. Crude mixtures were then purified by flash chromatography using pentane/diethyl ether as eluent.

## Supporting Information

File 1 Experimental part.

## References

[1] D’yakonov V A, Trapeznikova O A, de Meijere A, Dzhemilev U M (2014). Chem Rev.

[2] Salaün J, Bairds M S (1995). Curr Med Chem.

[3] Wong H N C, Hon M-Y, Tse C-W, Yip Y-C, Tanko J, Hudlicky T (1989). Chem Rev.

[4] Haym I, Brimble M A (2012). Org Biomol Chem.

[5] Orellana A, Nikolaev A (2016). Synthesis.

[6] Kulinkovich O G (2003). Chem Rev.

[7] Magrane J K, Cottle D L (1942). J Am Chem Soc.

[8] Imamoto T, Kamiya Y, Hatajima T, Takahashi H (1989). Tetrahedron Lett.

[9] Ito S, Shinokubo H, Oshima K (1998). Tetrahedron Lett.

[10] Rubottom G M, Lopez M I (1973). J Org Chem.

[11] Murai S, Aya T, Sonoda N (1973). J Org Chem.

[12] Du H, Long J, Shi Y (2006). Org Lett.

[13] Fontani P, Carboni B, Vaultier M, Maas G (1991). Synthesis.

[14] Pietruszka J, Widenmeyer M (1997). Synlett.

[15] Hussain M M, Li H, Hussain N, Ureña M, Carroll P J, Walsh P J (2009). J Am Chem Soc.

[16] Imai T, Mineta H, Nishida S (1990). J Org Chem.

[17] Benoit G, Charette A B (2017). J Am Chem Soc.

[18] Barluenga J, Suero M G, Pérez-Sánchez I, Flórez J (2008). J Am Chem Soc.

[19] Ukai K, Oshima K, Matsubara S (2000). J Am Chem Soc.

[20] Matsubara S, Ukai K, Fushimi H, Yokota Y, Yoshino H, Oshima K, Omoto K, Ogawa A, Hioki Y, Fujimoto H (2002). Tetrahedron.

[21] Cheng K, Carroll P J, Walsh P J (2011). Org Lett.

[22] Turro N J (1969). Acc Chem Res.

[23] Salaun J (1983). Chem Rev.

[24] Salaün J, Bennani F, Compain J C, Fadel A, Ollivier J (1980). J Org Chem.

[25] Stolle A, Ollivier J, Piras P P, Salaün J, de Meijere A (1992). J Am Chem Soc.

[26] Trost B M, Bogdanowicz M J (1973). J Am Chem Soc.

[27] Trost B M, Bogdanowicz M J (1973). J Am Chem Soc.

[28] Longone D T, Wright W D (1969). Tetrahedron Lett.

[29] Kulinkovich O G, Sviridov S V, Vasilevskii D A, Pritytskaya T S (1989). Zh Org Khim.

[30] Kulinkovich O G, Sviridov S V, Vasilevski D A (1991). Synthesis.

[31] Sviridov S V, Vasilevskii D A, Savchenko A I, Pritytskaya T S, Kulinkovich O G (1991). Zh Org Khim.

[32] Vasilevskii D A, Savchenko A I, Sviridov S V, Kulinkovich O G (1991). Zh Org Khim.

[33] Savchenko I A, Kulinkovich O G (1997). Zh Org Khim.

[34] Savchenko A I, Shevchuk T A, Kulinkovich O G (1999). Zh Org Khim.

[35] Lee J, Kim H, Cha J (1996). J Am Chem Soc.

[36] Epstein O L, Savchenko A I, Kulinkovich O G (1999). Tetrahedron Lett.

[37] Kananovich D G, Kulinkovich O G (2008). Tetrahedron.

[38] Lee J, Kang C H, Kim H, Cha J K (1996). J Am Chem Soc.

[39] Kasatkin A, Sato F (1995). Tetrahedron Lett.

[40] Corey E J, Rao S A, Noe M C (1994). J Am Chem Soc.

[41] Kulinkovich O G, Kananovich D G, Lopp M, Snieckus V (2014). Adv Synth Catal.

[42] Welch J G, Magid R M (1967). J Am Chem Soc.

[43] Lukina M Y, Rudavshevskaya T Y, Nesmeyanova O A (1970). Dokl Akad Nauk SSSR.

[44] Avezov I B, Bolesov I G, Levina R Y (1975). J Org Chem USSR.

[45] Moiseenkov A M, Czeskis B A, Semenovsky A V (1982). J Chem Soc, Chem Commun.

[46] Lehmkuhl H, Mehler K (1978). Justus Liebigs Ann Chem.

[47] Lehmkuhl H, Mehler K (1982). Liebigs Ann Chem.

[48] Richey H G, Watkins E K (1984). J Chem Soc, Chem Commun.

[49] Watkins E K, Richey H G (1992). Organometallics.

[50] Stoll A T, Negishi E-i (1985). Tetrahedron Lett.

[51] Rubin M, Rubina M, Gevorgyan V (2007). Chem Rev.

[52] Lautens M, Klute W, Tam W (1996). Chem Rev.

[53] Marek I, Simaan S, Masarwa A (2007). Angew Chem, Int Ed.

[54] Miege F, Meyer C, Cossy J (2011). Beilstein J Org Chem.

[55] Rubin M, Rubina M, Gevorgyan V (2006). Synthesis.

[56] Isaka M, Nakamura E (1990). J Am Chem Soc.

[57] Delaye P-O, Didier D, Marek I (2013). Angew Chem, Int Ed.

[58] Roy S R, Didier D, Kleiner A, Marek I (2016). Chem Sci.

[59] Liao L-a, Fox J M (2002). J Am Chem Soc.

[60] Nakamura M, Hirai A, Nakamura E (2000). J Am Chem Soc.

[61] Liu X, Fox J M (2006). J Am Chem Soc.

[62] Krämer K, Leong P, Lautens M (2011). Org Lett.

[63] Rubin M, Gevorgyan V (2004). Synthesis.

[64] Baird M S, de Meijere A (1996). Carbocyclic Three-Membered Ring Compounds.

[65] Petiniot N, Anciaux A J, Noels A F, Hubert A J, Teyssié P (1978). Tetrahedron Lett.

[66] Panne P, Fox J M (2007). J Am Chem Soc.

[67] Díaz-Requejo M M, Mairena M A, Belderrain T R, Nicasio M C, Trofimenko S, Pérez P J (2001). Chem Commun.

[68] Lipshutz B H, Wilhelm R S (1982). J Am Chem Soc.

[69] Lipshutz B H, Kozlowski J, Wilhelm R S (1982). J Am Chem Soc.

[70] Lipshutz B H, Wilhelm R S, Floyd D M (1981). J Am Chem Soc.

[71] Minko Y, Marek I (2014). Org Biomol Chem.

[72] Boche G, Lohrenz J C W (2001). Chem Rev.

[73] Müller E, Töpel T (1939). Ber Dtsch Chem Ges B.

[74] Boche G, Möbus K, Harms K, Lohrenz J C W, Marsch M (1996). Chem – Eur J.

[75] Möller M, Husemann M, Boche G (2001). J Organomet Chem.

[76] Warner P, Lu S-L (1976). J Org Chem.

[77] Surry D S, Spring D R (2006). Chem Soc Rev.

[78] Simaan M, Delaye P-O, Shi M, Marek I (2015). Angew Chem, Int Ed.

[79] Minko Y, Pasco M, Lercher L, Botoshansky M, Marek I (2012). Nature.

[80] Minko Y, Pasco M, Lercher L, Marek I (2013). Nat Protoc.

[81] Davies H M L, Lee G H (2004). Org Lett.

[82] Marek I, Simaan S, Masarwa A (2008). Angew Chem, Int Ed.

[83] Dian L, Müller D S, Marek I (2017). Angew Chem, Int Ed.

[84] Parra A, Amenós L, Guisán-Ceinos M, López A, García Ruano J L, Tortosa M (2014). J Am Chem Soc.

[85] Müller D S, Marek I (2015). J Am Chem Soc.

[86] Panek E J, Kaiser L R, Whitesides G M (1977). J Am Chem Soc.

[87] DeBergh J R, Spivey K M, Ready J M (2008). J Am Chem Soc.

[88] Zhang D, Ready J M (2005). Org Lett.

[89] Simaan M, Marek I (2018). Angew Chem, Int Ed.

[90] Sommer H, Marek I (2018). Chem Sci.

[91] Dian L, Marek I (2018). Angew Chem, Int Ed.

[92] Müller D S, Werner V, Akyol S, Schmalz H-G, Marek I (2017). Org Lett.

