# Supplementary material for: Diastereo- and enantioselective preparation of cyclopropanol derivatives
Source: Beilstein J Org Chem. 2019 Mar 21;15:752–60. doi: 10.3762/bjoc.15.71 (PMC6444454; doi:10.3762/bjoc.15.71)
Supplement: File 1 — Experimental part. [file Beilstein_J_Org_Chem-15-752-s001.pdf]

BEILSTEIN JOURNAL OF ORGANIC CHEMISTRY

Supporting Information

for

Diastereo- and enantioselective preparation of cyclopropanol derivatives

Marwan Simaan and Ilan Marek

Beilstein J. Org. Chem. **2019,** *15,* 752–760. doi:10.3762/bjoc.15.71

Experimental part

License and Terms: This is a supporting information file under the terms of the Creative Commons Attribution License (http://creativecommons.org/licenses/by/4.0). Please note that the reuse, redistribution and reproduction in particular requires that the authors and source are credited.
The license is subject to the *Beilstein Journal of Organic Chemistry* terms and conditions: (https://www.beilstein-journals.org/bjoc)

Unless stated otherwise, all glasswares were flame dried under vacuum, and cooled under argon prior to use. All reactions were carried out under positive pressure of argon. Thin-layer chromatography (TLC) was performed using Merck© silica gel 60 F254 plates. Column chromatography was performed using Bio-Lab silica gel 60A (0.040–0.063mm). ^{1}H NMR and ^{13}C NMR spectra were recorded on a Bruker© spectrometers DPX200, AV300 or AVIII400, AV500 and AV600 using $CDCl_3$ (unless otherwise specified) as solvent. Chemical shifts are reported in parts per million (ppm) with respect to the residual solvent signal $CDCl_3$ (^{1}H NMR: δ = 7.24 ppm; ^{13}C NMR: δ = 77.16 ppm). Peak multiplicities are reported as follows: s = singlet, bs = broad singlet, d = doublet, t = triplet, dd = doublet of doublets, td = triplet of doublets, m = multiplet. app =apparent, "bs" stands for broad singlet and "ABsyst" for AB system. HPLC chromatograms were recorded using Agilent© 1100 Series line with CHIRAL PAK® AD-H, CHIRALCEL®IA, CHIRALCEL®OD, CHIRALCEL®OC-H and CHIRAL PAK® AZ-H. $[\alpha]_D$ data were recorded using SCHMIDT and HAENSCH© Unipol L1000. Melting points were performed using Stuart Scientific SMP10 series. Thin-layer chromatography (TLC) was conducted with E. Merck silica gel 60 F_{254} pre-coated plates (0.25 mm) and visualized by exposure to UV light (254 nm) or stained with anisaldehyde, phosphomolybdic acid, or potassium permanganate. Column chromatography was performed using Fluka silica gel 60 Å (40–63mm, 230–400 mesh). High-resolution mass spectra (HRMS) were obtained by the mass spectrometry facility at the Technion.

Reagents and materials

All solvents were purified and dried prior to use, dichloromethane was distilled from CaH_2, Ether and THF were dried from Pure-Solv® Purification System (Innovative Technology©). Copper cyanide, rhodium acetate dimer, rhodium prolinate dimer, $Cu(MeCN)_4PF_6$, (*R,S*)-JOSIPHOS), butyllithium (1.6 M in hexane), *tert* butyl hydroperoxide (5.5 M in nonane), chloroform-D were purchased from Aldrich or Tzamal Dchem. Copper cyanide, all alkyl Grignard reagents were prepared from the corresponding alkyl bromides. All cyclopropene carboxylates were prepared according to the know procedure [1-4]. All unfunctionalized cyclopropenes were prepared according to literature reports and were consistent with literature data [1,4-13].

General procedure for the preparation of cyclopropenyl methyl ethers (3a–d)

To a flame dried 3-necked round bottom flask equipped with a Teflon coated stirring bar, under argon atmosphere is added a suspension of NaH (1.5 equiv) in Et_2O (3 mL/mmol) at 0 °C. Then substituted cyclopropanol **2** (1 equiv) was added dropwise over a period of 2 min and the reaction mixture was allowed to stir at this temperature for an additional hour before introducting MeI (1.2 equiv). The reaction mixture was warmed to room temperature and stirred overnight. After that the reaction was completed (determined by TLC analysis of hydrolyzed aliquots), the reaction mixture was cooled in an ice bath and water was added. The reaction was extracted with Et_2O and dried over $MgSO_4$ to afford the final cyclopropenyl methyl ether **3a–d** as clear oil.

1-(Methoxymethyl)-2-propylcycloprop-2-en-1-yl) benzene (3a)

R_f = 0.6 (Hex/EtOAc = 90:10). Yield 70%. Colorless oil. ^{1}H NMR (400 MHz, $CDCl_3$): δ= 0.89 (t, *J* = 7.2 Hz, 3H), 1.49-1.54 (m, 2 H), 2.39 (t, *J* = 7.2 Hz, 2H), 3.29 (s, 3H), 3.67 ($d_{AB\ syst}$, *J* = 10.2 Hz, 1H), 3.80 ($d_{AB\ syst}$, *J* = 10.2 Hz, 1H), 6.68 (s, 1H), 7.05-7.21 (m, 5H); ^{13}C NMR (100 MHz, $CDCl_3$): δ = 14.0, 20.8, 27.0, 29.1, 58.5, 80.4, 102.4, 124,8, 125.1, 126.3, 128.0, 146.8. HRMS (ESI) calcd. for $C_{14}H_{19}O$ $[M^{+}H]^{+}$: 203.1436; found: 203.1414.

(2-Butyl-1-(methoxymethyl)cycloprop-2-en-1-yl)benzene (3b)

R_f = 0.7 (Hex/EtOAc = 90:10). Yield 70%. Colorless oil. ^{1}H NMR (400 MHz, $CDCl_3$): δ = 0.82 (t, *J* = 7.6 Hz, 3H), 1.27-1.33 (m, 2H), 1.49 (m,2H), 2.39-2.44 (m, 2H), 3.30 (s, 3H), 3.70 ($d_{AB\ syst}$, *J* = 10.4

Hz, 1H), 3.81 ($d_{AB\ syst}$, J = 10 Hz, 1H), 6.67 (s, 1H), 7.05-7.13 (m,1H), 7.13-7.21 (m, 4H); 13C NMR (100 MHz, $CDCl_3$): δ = 13.9, 22.5, 24.7, 29.2, 29.5, 58.6, 80.4, 102.3, 125.0, 125.1, 126.3, 128.0, 146.8. HRMS (ESI) calcd. for $C_{15}H_{21}O$ $[M^{+}H]^{+}$: 217.1592; found: 217.1580.

(*R*)-(2-Cyclohexyl-1-(methoxymethyl)cycloprop-2-en-1-yl)benzene (3c)
R_f = 0.7 (Hex/EtOAc = 90:10). Yield 75%. Colorless oil. ^{1}H NMR (400 MHz, $CDCl_3$): δ = 1.14-1.27 (m, 5H), 1.50-1.53 (m, 1H), 1.53-1.59 (m, 2H), 1.60-1.77 (m, 2H), 2.44-2.47 (bs, 1 H), 3.29 (s, 3H), 3.57 ($d_{AB\ syst}$, J = 10Hz, 1H), 3.90 ($d_{AB\ syst}$, J = 10Hz, 1H), 6.63 (s, 1H), 7.02-7.06 (m, 1H), 7.14-7.16 (m, 4H) ^{13}C NMR (100 MHz, $CDCl_3$): δ = 25.6, 25.6, 26.1, 29.5, 31.1, 31.1, 34.8, 58.5, 80.6, 100.7, 125.0, 126.3, 128.0, 128.7, 147.1; HRMS (ESI) calcd. for $C_{17}H_{23}O$ $[M^{+}H]^{+}$: 243.1749; found: 243.1729.
The enantiomerically enriched form was obtained with similar spectral data, $[\alpha]_D$: ($CHCl_3$, c = 2): 36.79°. *er* 93:7. *The enantiomeric ratio assigned to the title compound was based on the enantiomeric ratio found for compound (R)-(2-cyclohexyl-1-phenylcycloprop-2-en-1-yl)methanol.*

1-Hexyl-3-(methoxymethyl)-3-methylcycloprop-1-ene (3d)
R_f = 0.65 (Hex/EtOAc = 90:10). Yield 66%. Colorless oil. ^{1}H NMR (400 MHz, $CDCl_3$): δ = 0.82 (t, J = 7.2 Hz, 3H), 1.05 (s, 3H), 1.21-1.30 (m, 6H), 1.30-1.48 (m, 2H), 2.36 (t, J = 7.2 Hz, 2H), 3.23 (s, 5H), 6.64 (s, 1H); ^{13}C NMR (100 MHz, $CDCl_3$): δ = 14.2, 22.1, 22.6, 22.7, 25.8, 27.5, 29.1, 31.8, 58.4, 82.9, 108.4, 131.8. HRMS (ESI) calcd. for $C_{12}H_{23}O$ $[M+H]^{+}$: 183.1749; found: 183.1728.

General procedure for the carbocupration reaction of 3a,c with RCuCNLi
To a suspension of CuCN (1.5 equiv) in 8 ml of Et_2O was added alkyllithium dropwise at −35 °C (2 equiv.). The resulting mixture (pale yellow in case of MeLi and PhLi and dark brown in case of *n*-BuLi and *n*-HexLi) was allowed to stir for 30 min. Cyclopropene **3a–d** (1 equiv in 2 mL/mmol of Et_2O) was added at that temperature and the reaction mixture was stirred until TLC shows complete consumption of the starting material (eluent hexane/EtOAc 9:1 ca. 30 min). The reaction was then quenched with an aqueous solution of NH_4Cl/NH_4OH (2:1). The aqueous layer was extracted twice with EtOAc and the combined organic phases were washed with brine, dried over $MgSO_4$, filtered, and concentrated under reduced pressure. The crude mixtures were then purified by flash chromatography using pentane/diethyl ether as eluent.

1-(Methoxymethyl)-2-methyl-2-propylcyclopropyl)benzene (4a)
R_f = 0.6 (Hex/EtOAc = 90:10). Yield 67%. Colorless oil, ^{1}H NMR (400 MHz, $CDCl_3$): δ = 0.36-0.47 (m, 1H), 0.70 (t, J = 7.2 Hz, 3H), 1.11 (m, 1H), 1.17-1.20 (m, 4H), 1.25-1.45 (m, 1H), 3.20 (s,3H), 3.60 (m,2H), 7.14-7.16 (m, 1H), 7.19-7.30 (m, 4H); ^{13}C NMR (100 MHz, $CDCl_3$): δ = 15.0, 17.9, 20.0, 24.2, 27.3, 32.2, 44.5, 59.9, 83.1, 125.6, 128.5. 130.1, 144.3 HRMS (ESI) calcd. for $C_{15}H_{23}O$ $[M^{+}H]^{+}$: 219.1799; found: 219.1764.

2-Butyl-1-((methoxymethyl)-2-propylcyclopropyl)benzene (4b)
R_f = 0.6 (Hex/EtOAc = 90:10). Yield 72%. Colorless oil. ^{1}H NMR (400 MHz, $CDCl_3$): δ = 0.35-0.45 (m, 1H), 0.58 (t, J = 7.2 Hz, 3H), 0.88 (t, J = 7.2 Hz, 3H), 1.01-1.03 (m, 1H), 1.20-1.22 (m, 3H), 1.30-1.33 (m, 3H), 1.44-1.50 (m, 2H), 1.55-1.59 (m, 2H), 3.23 (s, 3H), 3.74 (bs, 2H), 7.12-7.30(m, 5H), ^{13}C NMR (100 MHz, CDCl3): δ = 14.1, 14.7, 18.4, 21.0, 27.5, 31.2, 39.2, 41.6, 60.0, 82.9, 126.2, 127.8, 129.6, 144.1 . HRMS (ESI) calcd. for $C_{18}H_{29}O$ $[M^{+}H]^{+}$: 261.2218; found: 261.2210.

(1-(Methoxymethyl)-2-propylcyclopropane-1,2-diyl)dibenzene (4c)
R_f = 0.54 (Hex/EtOAc = 90:10). Yield 62%. Colorless oil, ^{1}H NMR (400 MHz, $CDCl_3$): δ = 0.39 (m, 1H), 0.44-0.46 (m, 1H), 0.65 (t, J = 7.2 Hz, 3H), 1.01-1.03 (m, 1H), 1.12-1.15 (m, 2H), 1.30-1.35 (m,

1H), 3.02 (s, 3H), 3.25 ($d_{AB\ syst}$, J = 10.0 Hz, 1H), 3.70 ($d_{AB\ syst}$, J = 10.0 Hz, 1H), 3.94, 7.25-7.33 (m, 2H), 7.35-7.42 (m, 8H); ^{13}C NMR (100 MHz, $CDCl_3$): δ = 14.7, 18.1, 23.3, 28.5, 32.9, 42.3, 60.1, 82.7, 126.6, 126.8, 128.4, 128.6, 130.1, 139.5, 141.3 . HRMS (ESI) calcd. for $C_{20}H_{25}O$ $[M+H]^+$: 281.1905; found: 281.1920.

(2-Cyclohexyl-1-(methoxymethyl)-2-methylcyclopropyl)benzene (4d)
R_f = 0.6 (Hex/EtOAc = 90:10). Yield 62%. Colorless oil. ^{1}H NMR (400 MHz, $CDCl_3$): δ = 0.060-0.115 (m, 1H), 0.56-0.60 (m,1H), 0.90-0.95 (m,3H), 1.01 (m, 1H), 1.05 (s, 3H), 1.08-1.11 (m, 2H), 1.45-1.48 (m, 2H), 1.52-1.58 (m, 2H), 3.20 (s, 3H), 3.70 ($d_{AB\ syst}$, J = 10Hz, 1H), 3.81 ($d_{AB\ syst}$t, J = 10Hz, 1H), 7.15-7.18 (m, 1H), 7.21-7.24 (m, 2H), 7.27-7.29 (m, 2H); ^{13}C NMR (100 MHz, $CDCl_3$): δ = 7.6, 26.7, 26.8, 26.9, 28.3, 29.5, 35.5, 40.0, 42.0, 61.0, 75.5, 128.3, 129.0, 129.5, 141.8. HRMS (ESI) calcd. for $C_{18}H_{27}O$ $[M+H]^+$: 259.2061; found: 259.2062.

(2-Butyl-2-cyclohexyl-1-(methoxymethyl)cyclopropyl)benzene (4e)
R_f = 0.6 (Hex/EtOAc = 90:10). Yield 60%. Colorless oil. ^{1}H NMR (400 MHz, $CDCl_3$): δ = 0.03-0.04 (m, 1H), 0.42-0.44 (m, 1H), 1.01 (t, J = 7.2Hz, 3H), 1.05-1.06 (m, 3H), 1.08-1.10 (m, 1H) 1.13-1.16 (m, 2H), 1.38-1.51 (m, 5H), 1.55-1.62 (m, 5H), 3.11 (s, 3H), 3.77 ($d_{AB\ syst}$, J = 9.2Hz, 1H), 3.85 ($d_{AB\ syst}$, J = 9.2Hz, 1H), 7.15-7.19 (m, 1H), 7.25-7.30 (m, 4H); ^{13}C NMR (100 MHz, $CDCl_3$): δ = 14.6, 23.2, 24.5, 26.8, 27.1, 27.5, 30.8, 31.2, 32.2, 40.5, 41.5, 44.8, 61.9, 78.2, 127.3, 128.8, 131.0, 141.6. HRMS (ESI) calcd. for $C_{21}H_{33}O$ $[M+H]^+$: 301.2531; found: 301.2540.

General procedure for the combined carbocupration/oxidation sequence
The reaction was performed on a 1 mmol scale. To a suspension of CuCN (2 equiv) in 8 mL of Et_2O was added alkyllithium dropwise at −35 °C (2 equiv./2mmol). The resulting mixture (pale yellow in case of MeLi and PhLi and dark brown in case of *n*-BuLi and *n*-HexLi) was allowed to stir for 30 min. Cyclopropene **6** (1 equiv/1 mmol in 2 mL of Et_2O) was added at that temperature and the reaction mixture was stirred until TLC shows complete consumption of the starting material (eluent hexane/EtOAc 9:1 ca. 30 min). The oxenoid was prepared in a different flask by slowly adding *n*-BuLi (1.2 equiv) to a solution of *tert*-butyl hydroperoxide (2 equiv) in THF (5 mL/2 mmol) at −80 °C. After 30 min at −80 °C, the resulting *t*-BuOOLi was transferred to the organocopper dropwise at −78 °C via a cannula. The mixture (orange to brown) was stirred at this temperature until disappearance of the cyclopropylcopper species (followed by TLC, eluent hexane/EtOAc 9:1, ca. 30 min). The reaction was then quenched with an aqueous solution of NH_4Cl/NH_4OH (2:1). The aqueous layer was extracted twice with Et_2O and the combined organic phases were washed with brine, dried over $MgSO_4$, filtered, and concentrated under reduced pressure. Crude mixtures were then purified by flash chromatography using pentane/diethyl ether as eluent.

2,3-Dimethyl-2-phenylcyclopropan-1-ol (7a)
R_f = 0.25 (Hex/EtOAc = 90:10). Yield 65%. Colorless oil, ^{1}H (300 MHz, $CDCl_3$): δ = 0.90 (d, J = 6.2 Hz, 3H), 1.05-1.09 (m, 1H), 1.35 (s, 3H), 3.72 (d, J = 6.2 Hz, 1H), 7.20-7.30 (m, 4H); ^{13}C NMR (75 MHz, $CDCl_3$): δ = 15.1, 25.3, 32.3, 58.2, 125.6, 126.0, 128.2, 128.3, 150.4; HRMS (ESI) calcd. for $C_{14}H_{20}O$ $[M+H]^+$: 205.1592; found: 205.1594.

3-Butyl-2-methyl-2-phenylcyclopropan-1-ol (7b)
R_f = 0.2 (Hex/EtOAc = 90:10). Yield 62%. Colorless oil ^{1}H NMR (300 MHz, $CDCl_3$): δ = 0.85 (t, J = 7.2Hz, 3H), 0.97 (dt, J_1 =7.1Hz, J_2= 6.8Hz, 1H), 1.20-1.26 (m, 4H), 1.31-1.41 (m, 4H), 1.41-1.47 (m, 2H), 2.01 (bs, 1H), 3.53 (d, J = 7.2 Hz, 1H), 7.03-7.21 (m, 5H); ^{13}C NMR (75 MHz, $CDCl_3$): δ = 13.4, 14.3, 21.9, 22.9, 27.2, 28.7, 32.3, 57.9, 125.7, 127.2, 128.5, 147.9. HRMS (ESI) calcd. for $C_{14}H_{21}O$ $[M^+H]^+$: 205.1592; found: 205.1963.

3-Hexyl-2-methyl-2-phenylcyclopropan-1-ol (7c)

R_f = 0.2 (Hex/EtOAc = 90:10). Yield 60%. Colorless oil, ^{1}H NMR (300 MHz, $CDCl_3$): δ = 0.844 (t, *J* = 6.7 Hz, 3H), 0.94 (dt, J_1 =7.1Hz, J_2 = 6.6Hz, 1H), 1.27-1.41 (m, 9H), 1.41-1.44 (m, 4H), 1.81 (bs, 1H), 3.54 (d, *J* = 7.2 Hz, 1H), 7.07-7.19 (m, 5H); ^{13}C NMR (75 MHz, $CDCl_3$): δ = 13.5. 14.3, 22.3, 22.8, 27.3, 28.8, 29.5, 30.0, 32.0, 58.0, 125.7, 127.2, 128.5, 148.0. HRMS (ESI) calcd. for $C_{16}H_{25}O$ $[M^+H]^+$: 233.1905; found: 233.1902.

2-Methyl-2,3-diphenylcyclopropan-1-ol (7d)

R_f = 0.2 (Hex/EtOAc = 90:10). Yield 49%. Colorless oil, ^{1}H (400 MHz, $CDCl_3$): δ = 1.23 (s, 3H), 1.83 (bs, 1H), 2.32 (d, *J* = 8Hz, 1H), 3.92 (d, *J* = 8Hz, 1H), 7.14-7.24 (m, 2H), 7.25-7.33 (m, 8H); ^{13}C NMR (100 MHz, $CDCl_3$): δ = 15.7, 30.3, 32.2, 59.3, 126.2, 126.5, 127.4, 128.6, 128.7, 130.9, 135.6, 147.

3-Hexyl-2-methyl-2-(*p*-tolyl)cyclopropan-1-ol (7e)

R_f = 0.3 (Hex/EtOAc = 90:10). Yield 56%. Colorless oil, ^{1}H (300 MHz, $CDCl_3$): δ = 0.84 (t, *J* = 6.8 Hz, 3H), 0.94- 0.95 (m, 1H), 1.25-1.30 (m, 8H), 1.34-1.43 (m, 4H), 1.72 (bs, 1H), 2.23 (s, 3H), 2.53 (d, *J* = 5.4 Hz, 1H), 7-7.12 (m, 4H); ^{13}C NMR (75 MHz, $CDCl_3$): δ = 13.6, 14.3, 21.1, 22.3, 22.8, 27, 28.6, 29.5, 30, 32, 57.9, 127.1, 129.2, 135.3, 145.

3-Isopropyl-2-methyl-2-(*p*-tolyl)cyclopropan-1-ol (7f)

R_f = 0.25 (Hex/EtOAc = 90:10). Yield 53 %. Colorless oil, ^{1}H NMR (300 MHz, $CDCl_3$): δ = 0.78 (dd, J_1 = 7.2Hz, J_2 = 3.5Hz, 1H), 1.02 (d, *J* = 6.5Hz, 3H), 1.09 (d, *J* = 6.5Hz, 3H), 1.37 (s, 3H), 1.71 (bs,1H), 1.72-1.80 (m, 1H), 2.30 (s, 3H), 3.59 (d, *J* = 9Hz, 1H), 7.09 (m, 4H); ^{13}C NMR (75 MHz, $CDCl_3$): δ = 13.9, 21.1, 22.8, 23.0, 23.1, 27.4, 36.5 58.0, 127.3, 129.2, 135.3, 145.1HRMS (ESI) calcd. for $C_{14}H_{21}O$ $[M^+H]^+$: 205.1587; found: 205.1580

3-(*tert*-Butyl)-2-methyl-2-(*p*-tolyl)cyclopropan-1-ol (7g)

R_f = 0.3 (Hex/EtOAc = 90:10). Yield 50%. Colorless oil, ^{1}H (300 MHz, $CDCl_3$): δ = 0.68 (d, *J* = 9Hz, 1H), 1.10 (s, 9H), 1.44 (s,3H), 1.89 (bs, 1H), 2.21 (s, 3H), 3.64 (d, *J* = 9Hz, 1H), 6.98-7.07 (m, 3H); ^{13}C NMR (75 MHz, $CDCl_3$): δ = 15.5, 21.1, 28.7, 31.6, 32.4, 38.9, 59.5, 127.5, 129.2, 135.2, 147.

2-Methyl-3-phenyl-2-(*p*-tolyl)cyclopropan-1-ol (7h)

R_f = 0.2 (Hex/EtOAc = 90:10). Yield 57%. Colorless oil, ^{1}H (300 MHz, $CDCl_3$): δ = 1.31 (s, 3H), 1.98 (bs, 1H), 2.37-2.41 (m, 4H), 3.99 (d, *J* = 6 Hz, 1H), 7.16- 7.19 (m, 2H), 7.28-7.30 (m, 3H), 7.37-7.41 (m, 4H); ^{13}C NMR (75 MHz, $CDCl_3$): δ = 15.8, 21.1, 29.9, 32.1, 59.3, 126.5, 127.3, 128.6, 129.4, 130.9, 135.7, 135.8, 144.1. HRMS (ESI) calcd. for $C_{17}H_{18}O$ $[M+H]^+$: 239.1436; found: 239.1445.

2-Butyl-2,3-dimethyl-3-phenylcyclopropan-1-ol (7i)

R_f = 0.25 (Hex/EtOAc = 90:10). Yield 65%. Colorless oil, ^{1}H (400 MHz, $CDCl_3$): δ = 0.76 (t, *J* = 7.2 Hz, 3H), 0.76-0.93 (m, 1H), 1.11-1.20 (m, 5H), 1.20-1.40 (m, 6H), 1.40-1.43 (m, 2H), 3.12 (s, 1H), 7.14-7.17 (m, 3H), 7.19-7.27 (m, 2H) ; ^{13}C NMR (100 MHz, $CDCl_3$): δ =14.3, 17.5, 23.4, 24.5, 27.9, 29.4, 32.2, 32.5, 65.3, 126.1, 128.7, 130.6, 142.2.

2,3-Dimethyl-2,3-diphenylcyclopropan-1-ol (7j)

R_f = 0.20 (Hex/EtOAc = 90:10). Yield 56%. Colorless oil, ^{1}H (300 MHz, $CDCl_3$): δ = 0.84 (s, 3H), 1.08 (s, 3H), 2.14 (bs, 1H), 3.73 (s, 1H), 7.09-7.13 (m, 2H), 7.14-7.28 (m, 8H); ^{13}C NMR (75 MHz, $CDCl_3$): δ= 20.4, 26.7, 33.4, 35.4, 63.1, 126.2, 126.3, 128.6, 128.8, 129.1, 130.5, 140.8, 144; HRMS (ESI) calcd. for $C_{17}H_{19}O$ $[M+H]^+$: 239.1428; found: 239.1433.

2,3-Dimethyl-2-phenyl-3-propylcyclopropan-1-ol (7k)

R_f = 0.25 (Hex/EtOAc = 90:10). Yield 70%. Colorless oil, ^{1}H (400 MHz, $CDCl_3$): δ = 0.42-0.43 (m, 1H), 0.61 (t, *J*= 7.2 Hz, 3H), 0.97-0.98 (m, 1H), 1.06 (s, 3H), 1.17-1.24 (m, 5H), 2.10 (bs, 1H), 3.47 (s, 1H), 7.07-7.20 (m, 5H); ^{13}C NMR (100 MHz, $CDCl_3$): δ = 11.2, 14.3, 16.7, 19.7, 27.9, 33.3, 38.7, 60.9, 125.8, 128.3, 128.9, 145.2; HRMS (ESI) calcd. for $C_{14}H_{21}O$ $[M+H]^+$: 205.1592; found: 205.1596.

2-Butyl-3-methyl-3-phenyl-2-propylcyclopropan-1-ol (7l)

R_f = 0.3 (Hex/EtOAc = 90:10). Yield 70%. Colorless oil, ^{1}H (400 MHz, $CDCl_3$): δ = 0.05-0.07 (m, 1H), 0.63 (t, *J* = 7.2 Hz, 3H), 0.89 (t, *J* = 7.2 Hz, 3H), 1.18-0.134 (m, 9H), 1.44-1.46 (m, 3H), 2.08 (bs, 1H), 3.44 (s, 1H), 7.06-7.20 (m, 5H); ^{13}C NMR (100 MHz, $CDCl_3$): δ= 14.3, 14.3, 17.1, 19.6, 23.5, 23.8, 28.6, 31.6, 33.9, 35.5, 62.1, 125.8, 128.3, 129.1, 145.3; HRMS (ESI) calcd. for $C_{17}H_{27}O$ $[M+H]^+$: 247.2062; found: 247.2102.

2-Butyl-2-hexyl-3-methyl-3-phenylcyclopropan-1-ol (7m)

R_f = 0.3 (Hex/EtOAc = 90:10). Yield 60%. Colorless oil, ^{1}H (400 MHz, $CDCl_3$): δ = 0.90-0.12 (m, 1H), 0.72 (t, *J* = 7.2 Hz, 3H), 0.89 (t, *J* = 7.2 Hz, 3H), 0.91-1.31 (m, 8H), 1.31-1.45 (m, 3H), 2.18 (bs, 1H), 3.44 (s, 1H), 7.06-7.19 (m, 5H) ; ^{13}C NMR (100 MHz, $CDCl_3$): δ = 14.1, 14.3, 17.1, 22.7, 23.5, 23.8, 26.4, 28.7, 29.6, 31.7, 32, 33.3, 34.1, 62, 125.7, 128.3, 129.1, 145.3. HRMS (ESI) calcd. for $C_{20}H_{32}O$ $[M+H]^+$: 288.2453; found: 288.2450.

2-Hexyl-2,3-dimethyl-3-phenylcyclopropan-1-ol (7n)

R_f = 0.3 (Hex/EtOAc = 90:10). Yield 68%. Colorless oil, ^{1}H (400 MHz, $CDCl_3$): δ = 0.44-0.48 (m, 1H), 0.72 (t, *J*= 7.2 Hz, 3H), 0.96-1.11 (m, 12H), 1.11-1.24 (m, 4H), 2.20 (bs, 1H), 3.46 (s, 1H), 7.06-7.08 (m, 1H), 7.12-7.19(m, 4H); ^{13}C NMR (100 MHz, $CDCl_3$): δ = 11.2, 14.1, 16.6, 22.6, 26.4, 27.9, 29.5, 31.8, 33.4, 36.5, 60.7, 125.7, 128.2, 128.9, 145.1; HRMS (ESI) calcd. for $C_{17}H_{26}O$ $[M+H]^+$: 247.2062; found: 247.2100.

2-Butyl-3-methyl-2-propyl-3-(*p*-tolyl)cyclopropan-1-ol (7o)

R_f = 0.3 (Hex/EtOAc = 90:10). Yield 52%. Colorless oil, ^{1}H (300 MHz, $CDCl_3$): δ = 0.65 (t, *J* = 6 Hz, 3H), 0.92 (t, *J* = 6 Hz, 3H), 1.19-1.36 (m, 10H), 1.44-1.45 (m, 3H), 1.82 (bs, 1H), 2.24 (s, 3H), 3.42 (s,1H), 7.01 (m, 4H); ^{13}C NMR (75 MHz, $CDCl_3$): δ = 14.4, 17.2, 19.6, 21.2, 23.5, 23.7, 28.6, 31.4, 33.5, 35.4, 62.2, 129.1, 135.2, 142.2; HRMS (ESI) calcd. for $C_{18}H_{29}O$ $[M+H]^+$: 261.2218; found: 261.2210.

2-Hexyl-3-methyl-2-propyl-3-(*p*-tolyl)cyclopropan-1-ol (7p)

R_f = 0.3 (Hex/EtOAc = 90:10). Yield 47%. Colorless oil, ^{1}H (300 MHz, $CDCl_3$): δ = 0.05-0.09 (m, 1H), 0.65 (t, *J* = 7 Hz, 3H), 0.84 (t, *J* = 7 Hz, 3H), 1.18-1.27 (m, 13H), 1.41-1.48 (m, 3H), 1.97 (bs, 1H), 2.23 (s, 3H), 3.42 (s, 1H), 7.00 (s, 4H) ; ^{13}C NMR (75 MHz, $CDCl_3$): δ = 14.3, 14.4, 17.2, 19.6, 21.2, 22.9, 24, 26.4, 30.2, 31.5, 32.1, 33.5, 35.4, 62.2, 128.9, 129.1, 135.2, 142.2. $C_{20}H_{33}O$ $[M^+H]^+$: 289.2531; found: 289.2529.

2-Butyl-3-(4-chlorophenyl)-3-methyl-2-propylcyclopropan-1-ol (7q)

R_f = 0.25 (Hex/EtOAc = 90:10). Yield 51%. Colorless oil, ^{1}H (600 MHz, $CDCl_3$): δ = 0.12 (m, 1H), 0.96 (t, *J* = 6 Hz, 3H), 1.26 (t, *J* = 6 Hz, 3H), 1.29-1.41 (m, 9H), 1.51-1.53 (m, 3H), 1.97 (bs, 1H), 3.49 (s, 1H), 7.12- 7.13 (d, *J*= 6Hz, 2H), 7.23-7.25 (d, *J*= 6Hz, 2H) ; ^{13}C NMR (150 MHz, $CDCl_3$): δ = 14.3, 14.4, 16.9, 23.5, 23.7, 28.6, 31.7, 33.4, 35.5, 62.1, 128.5, 130.5, 131.4, 143.8. HRMS (ESI) calcd. for $C_{17}H_{26}ClO$ [M]: 280.1570; found: 280.1598.

2-(4-Chlorophenyl)-3-hexyl-2-methyl-3-propylcyclopropan-1-ol (7r)

R_f = 0.25 (Hex/EtOAc = 90:10). Yield 48%. Colorless oil, ^{1}H (300 MHz, $CDCl_3$): δ = 0.05-012 (m, 1H), 0.70 (t, *J* = 6Hz, 3H), 0.89 (t, *J* = 6Hz, 3H), 1.22-1.33 (m, 14H), 1.48-1.49 (m, 3H), 1.72 (bs, 1H), 3.46 (s, 1H); 7.09 ($d_{AB\ sys}$, *J* = 6Hz, 2H), 7.21 ($d_{AB\ sys}$, *J* = 6Hz, 2H); ^{13}C NMR (75 MHz, $CDCl_3$): δ = 14.3, 14.4, 17, 19.6, 22.9, 24, 26.4, 30.2, 31.8, 32, 33.4, 35.5, 62.1, 128.6, 130.5, 131.4, 143.8. HRMS (ESI) calcd. for $C_{19}H_{30}ClO$ [M]: 309.1985; found: 309.1974.

References

1. Delaye P-O.; Didier D.; Marek, I. *Angew. Chem. Int. Ed.*, **2013**, 52, 5333.
2. Li, C.; Zeng, Y.; Wang, J. *Tetrahedron Lett*. **2009**, 50, 2956.
3. Tarwade, V.; Liu, X.; Yan, N.; Fox, J. M. *J. Am. Chem. Soc*. **2009**, 131, 5382.
4. Uehara, M.; Suematsu, H.; Katsuki, T. *J. Am. Chem. Soc*. **2011**, *133*, 170.
5. Phan, D. H. T.; Kou, K. G. M.; Dong, V. M. *J. Am. Chem. Soc.*, **2010**, 132, 16354.
6. Kremer, K.; Leong, P.; Lautens, M. *Org. Lett.* **2011**, 13, 819.
7. Sherrill, W.M.; Kim, R.; Rubina, M. *Tetrahedron*, **2008**, 64, 8610.
8. Smith, M. A.; Richey. H. G. Jr, *Organometallics*, **2007**, *26*, 609.
9. Chen, W.; Tao, H.; Huang, W.; Wang, G.; Li, S.; Cheng, X.; Li, G. *Chem. Eur. J*. **2016**, *22*, 9546.
10. Fort, D. A.; Woltering, T. J.; Nettekoven, M.; Knust, H.; Bach, T. *Chem. Commun.*, **2013**, **49**, 2989.
11. Pratsch, G.; Overman, L. E. *J. Org. Chem.*, **2015**, 80, 11388.
12. Rubina, M.; Rubin, M.; Gevorgyan, V. *J. Am. Chem. Soc.*, **2002**, *124*, 11566.
